# THE ROLE OF PARENTAL EDUCATION AND ADVERSE CHILDHOOD EXPERIENCES IN COGNITIVE FUNCTION AMONG DIVERSE OLDER ADULTS

**DOI:** 10.1093/geroni/igae098.0888

**Published:** 2024-12-31

**Authors:** Regina Wright

**Affiliations:** University of Delaware, Newark, Delaware, United States

## Abstract

An expansive body of research suggests older adults’ cognitive abilities are linked to a number of factors across the lifespan, including socioeconomic status and physical health. Early life experiences have emerged as an important predictor of cognitive function, as researchers identify long-term trajectories linking factors such as early life trauma and poverty to poor outcomes; however, much of these findings have been limited to largely homogenous study samples. Therefore, we examined the cross-sectional relationships between parental education, adverse childhood experiences (ACEs), and cognitive function in a diverse sample of community-dwelling older adults. Data were analyzed from 165 older adults (mean age = 68.48y, 33% male, 41% African American), free of major disease, who completed a battery of psychosocial and neuropsychological tests and a sociodemographic assessment, and underwent a physical screening in two study visits. We found no significant associations between ACEs and cognitive performance. However, linear regression analyses, adjusting for age, sex, race, education, and ACEs, showed a statistically significant association between maternal education and short-term verbal (p=.007) and visuospatial memory (p=.003) performance, and paternal education and long-term verbal memory performance(p=.029). Results further showed that maternal education was a stronger influence on cognitive performance than paternal education. Findings suggest having parents with greater educational attainment, and in particular a mother with greater educational attainment, is related to better cognitive performance in late life, independently of participant education or ACEs. This presentation will explore plausible pathways for intergenerational transmission of cognitive resilience.

